# The Inspector in Excelsis

**Published:** 1895-08-31

**Authors:** 


### THE INSPECTOR "IN EXCELSIS."

This is surely an age of inspection, and the inspector triumphs all along the line, for is he not now making an onslaught on the British householder's last stronghold? "Every man's house is his castle," has long been the Englishman's boast, but Dr. Spottiswoode Cameron, the energetic medical officer of health for Leeds, would not admit that anyone should be allowed to rule his house in his own way, or even to dwell in a house unless it comes up to modern sanitary standards. At present it is usual for the sanitary inspector to make his investigations only when complaints of nuisances are made, or when the occurrence of some infectious disease has been reported; but is it not absurd, he says, to wait for this? Is it not our duty to visit all houses, and ascertain beforehand whether the conditions there present are such as to favour or destroy the health of those who dwell in them? He advocates regular systematic inspection of every house, and, indeed, he shows good reason for his proposal. However well houses are built, and however properly the sanitary appliances are arranged they soon get out of repair, and no such forethought in passing plans or in supervising the erection of buildings as a sanitary authority may exercise can prevent these self-same houses becoming in a comparatively short time unhealthy dwellings. In default of periodical inspection the authority have no means of becoming aware of this deterioration until the occurrence of some form of infectious disease, or of some nuisance so great that the tenant himself complains and brings the matter to their notice. This is indeed locking the door after the steed is stolen, and Dr. Cameron does but desire to see that done regularly and in an orderly fashion which at present generally takes place either when sickness is in the house or when we are driven to ask for it by the occurrence of a nuisance in our midst.

In regard to the impossibility of judging as to the real sanitary condition of a house without a complete inspection, Dr. Cameron instances a row of houses which had been inspected in consequence of the occurrence of a case of diphtheria in one of them. These were modern houses, built under modern bye-laws, and carefully inspected when built, with every waste-pipe cut off from the sewer, and all sanitary conveniences outside the dwelling. Yet on investigation it was found that a strong-smelling chemical introduced into the sewer penetrated into the houses in seven cases out of ten, showing that, notwithstanding all the care which had been exercised in their erection only a few years before, a connection had become established between the air of the sewer and the air of a large proportion of the houses. Whether this resulted from the ravages of rats, porosity of soil, breaking of drain-pipes by traction engines or steam-rollers, or otherwise, does not matter; the fact, however, serves to show that the mere building of houses in a proper way gives no guarantee that they will keep right. But in many houses sanitary appliances have no pretension to being in any way up to date. In a recent inspection of 100 contiguous houses all more or less recently built, but not modern as in the last example, it was found that in one-third or more of them the wastes were not cut off from the drains, and that in 60 per cent. of these the smell of chemicals introduced into the sewer as a test permeated the houses themselves, thus making it evident that, however healthy the inhabitants might think themselves, they were living amid conditions sure to aggravate and intensify any illness which might befall them. Doubtless when illness arose, if it happened to take the form of one of the notifiable diseases, the sanitary inspector would enter and discover the deficiencies; but surely it is to take a very narrow view of sanitary science to look upon zymotic diseases as the only ones which are influenced for evil by defective health surroundings. Medical officers know, but the public is slow to recognise, how largely what are called general and even constitutional diseases have their character changed by the conditions amid which they occur. To wait before investigating this important factor in disease production until the malady occurs in a specified form is like refusing to send for the fire-engines till the conflagration bursts out of the windows. It should be widely recognised that all insanitary conditions are bad for all diseases, besides being entirely responsible for many; and if it be also understood that without systematic and repeated inspection it is impossible to know whether such insanitary conditions exist in our houses or not, we shall not be backward in agreeing with Dr. Cameron's recommendation. To clinch his argument, he states that in a town in which systematic inspection had been made, while the death-rate for five years preceding the introduction of this system had been 22·3 per 1,000, it had for the last five years been only 18·6—an improvement of more than 16 per cent.

